# The distinct hepatic metabolic profile and relation with impaired liver function in congenital isolated growth hormone-deficient rats

**DOI:** 10.1530/EC-23-0462

**Published:** 2024-04-04

**Authors:** Xiaonan Guo, Wenjing Hu, Xiaorui Lyu, Hanyuan Xu, Huijuan Zhu, Hui Pan, Linjie Wang, Hongbo Yang, Fengying Gong

**Affiliations:** 1 Key Laboratory of Endocrinology of National Health Commission, Department of Endocrinology, Peking Union Medical College Hospital, Chinese Academy of Medical Sciences and Peking Union Medical College, Beijing, China

**Keywords:** growth hormone deficiency, insulin-like growth hormone 1, liver function, hepatic metabolomics

## Abstract

**Objective:**

Patients with growth hormone deficiency (GHD) with inadequate growth hormone levels are often correlated with nonalcoholic fatty liver disease (NAFLD). However, the potential mechanism of how GHD influences liver function remains obscure. In the present study, we aim to perform hepatic metabolomics in Lewis dwarf rats, which were the standard congenital isolated GH-deficient rat, to evaluate the characterizations of hepatic metabolic profiles and explore their relations with liver functions.

**Methods:**

Lewis dwarf homozygous (dw/dw) rats at 37 weeks (five females and five males), and Lewis dwarf heterozygous (dw/+) rats at 37 weeks (five females and five males) were analyzed in our study. Body lengths and weights, liver weights, serum alanine transaminase (ALT), and serum aspartate transaminase (AST) were measured. ELISA and RT-qPCR were used to assess IGF-1 levels in serum and liver, respectively. The non-targeted metabolomics was performed in the livers of dw/+ and dw/dw rats. Differential metabolites were selected according to the coefficient of variation (CV), variable importance in the projection (VIP) > 1, and *P* < 0.05. Hierarchical clustering of differential metabolites was conducted, and the KEGG database was used for metabolic pathway analysis.

**Results:**

The body weights, body lengths, liver weights, and IGF-1 levels in the serum and liver of dw/dw rats were significantly decreased compared with dw/+ rats. Dw/dw rats exhibited more obvious hepatic steatosis accompanied by higher serum ALT and AST levels. Hepatic metabolomics showed that a total of 88 differential metabolites in positive ion mode, and 51 metabolites in negative ion mode were identified. Among them, lysophosphatidylcholine (LPC) 16:2, LPC 18:3, LPC 22:6, fatty acid esters of hydroxy fatty acids (FAHFA)18:1 were significantly decreased, while palmitoyl acid, dehydrocholic acid, and 7-ketolithocholic acid were significantly increased in dw/dw rats compared with dw/+ rats. These seven differential metabolites were significantly associated with phenotypes of rats. Finally, KEGG pathway analysis showed that the arginine and proline metabolism pathway and bile secretion pathway were mainly clustered.

**Conclusion:**

Lewis dw/dw rats with congenital isolated growth hormone deficiency (IGHD) showed liver steatosis and abnormal liver function, which could be potentially associated with the distinctive hepatic metabolic profiles.

## Introduction

Growth hormone (GH) is a significant regulator of growth and metabolism (1). In the liver, GH stimulates the synthesis and secretion of insulin-like growth factor 1 (IGF-1) (2). The GH/IGF-1 axis can regulate hepatic glucose and lipid metabolism, including glucose production, the uptake and storage of lipids, and the secretion of triglycerides (3). Growth hormone deficiency (GHD) is a relatively uncommon endocrine disease caused by a primary deficiency of GH and a secondary deficiency of IGF-1 (1). Several studies have shown that congenital GHD is associated with an increased risk of metabolic syndromes, such as nonalcoholic fatty liver disease (NAFLD), non-alcoholic steatohepatitis (NASH), and hyperlipidemia (4, 5, 6). For instance, a study of 22 congenital isolated GHD (IGHD) patients exhibited increased glutamic oxaloacetic transaminase (AST) levels, higher liver hyperechogenic pattern scores, and a higher prevalence of NAFLD compared with the control groups (4). Another study performed on 11 congenital IGHD patients showed that liver right or left lobes were significantly lower than the control groups but were similar after adjusting for body weight (5). Therefore, there is a significant need to further analyze the liver situation of congenital IGHD patients.

Metabolomics has been used in the comprehensive profiling of small molecular metabolites (<1 kDa) in cells, tissues, and whole organisms (7). It has been roughly divided into targeted metabolomics and untargeted metabolomics. In the targeted metabolomics, specific compounds are quantified and compared to established reference ranges. In untargeted metabolomics, all detectable metabolites are analyzed without any prior metabolic hypothesis and compared between groups of samples (7, 8). Accumulating studies have performed metabolomics in the serum, plasma, urine, or kidney of patients with GHD to further understand the mechanisms underlying the development and progression of GHD (8, 9, 10). For instance, serum metabolomics performed in patients with AGHD from Sweden showed nine metabolites, such as threnodic acid and cysteine, were significantly different when compared with controls, which can help to better differentiate patients with GHD from controls (8). A urine metabolomics study in GHD patients reported distinct profiles when compared with healthy controls, which might provide new markers for the diagnosis of GHD (9). So far, no attempt to perform hepatic analysis has been made in patients with congenital GHD. As we all know, the liver is the metabonomic hub of the body, and the alterations of the metabolome in the liver are crucial for uncovering the mechanism of hepatic function. Thus, it is essential to perform untargeted hepatic metabolomics in patients with congenital GHD to explore the potential mechanisms of alterations in liver function.

Lewis dwarf homozygous (dw/dw) rats have been widely regarded as a classical model of congenital isolated GHD, which exhibited similar phenotypes observed in patients with childhood-onset adult growth hormone deficiency (CO AGHD) (11, 12, 13, 14, 15, 16). It was first reported with significant reductions in pituitary GH synthesis and secretion in 1988 (16). The pituitary GH levels were about 10% of normal in males and 6% in females (16). Notably, their other anterior pituitary hormones, including prolactin, thyrotropin, luteinizing hormone, and adrenocorticotropic hormone, were within the normal ranges (16). Previous studies have shown that Lewis dw/dw rats exhibited severe growth retardation, disturbed lipid and glucose metabolism (17, 18, 19). However, no study has reported the hepatic function of Lewis dw/dw rats.

Therefore, we aimed to apply untargeted hepatic metabolomics in Lewis dw/dw rats to identify differential metabolites and metabolic pathways and explore their relationship with hepatic function.

## Materials and methods

### Animals

Lewis homozygous (dw/dw) rats were provided by Professor Michael J Waters, University of Queensland, Australia. Lewis wild-type (WT) rats were purchased from Beijing Vital River Laboratory Animal Technology Co., Ltd. (Beijing, China). Lewis heterozygote (dw/+) rats were generated by mating Lewis dw/dw and Lewis WT rats. Lewis dw/dw rats at 37 weeks (five females and five males), and Lewis dw/+ rats at 37 weeks (five females and five males) were analyzed in our study. The rats were housed in a standard 12h light/dark cycle environment with sufficient water and food (SF; 10% kcal fat, H10010, Beijing HFK Bioscience Co., Ltd., Beijing, China). The rats were in narcotism by injecting 2.5% tribromoethanol into the cavum abdominis (15 mL/kg). All rats were weighed before sacrifice. The body lengths were measured from the nose’s tip to the tail’s tip after sacrifice. The intact livers were collected and then weighed. The ethics committee of Peking Union Medical College Hospital approved the animal experiment protocols (XHDW-2021-035).

### Samples collection

All blood samples were collected from the abdominal aorta and immediately centrifuged at 3000 ***g*** for 10 min at 4°C under anesthesia before sacrifice. Serum IGF-1 levels were measured with ELISA kits (Cloud-Clone Corp., Wuhan, China) according to the manufacturer's protocols. Serum alanine transaminase (ALT) and aspartate transaminase (AST) levels were measured by an automatic biochemistry analyzer (Cobas Integra 400 plus, Roche Kit).

### Liver histologic analysis

A portion of liver samples (1 cm^3^) was fixed in 10% formalin, dehydrated, and embedded in paraffin. Sections (3 µm) were stained with hematoxylin and eosin (H&E) using standard procedures. Images were obtained using a digital camera (Nikon DS-U3, Japan). The remaining liver was frozen in liquid nitrogen and then stored at −80°C in Clinical Biobank, Peking Union Medical College Hospital, Chinese Academy of Medical Sciences (Beijing, China) until further experiments.

### Reverse transcription quantitative polymerase chain reaction

The total RNA of the liver was extracted with the total RNA Kit II (R6934, Omega Bio-tek, Norcross, GA, USA) following the manufacturer’s protocol. Complementary DNA (cDNA) was synthesized with 0.5 μg of total RNA using the PrimeScriptTM RT reagent Kit with the gDNA Eraser (RR047A, TaKaRa) under the following conditions: 42°C for 2 min, 37°C for 15 min, and 85°C for 5 s. The expression of IGF-1 was measured using the TB Green® Premix Ex Taq II (RR820A, TaKaRa) in the ABI7500 PCR system (Applied Biosystems). In addition, glyceraldehyde 3-phosphate dehydrogenase (GAPDH) was used as an internal control. The primer sequences for IGF-1 and GAPDH were as follows: IGF-1: forward 5′-AAGCCTACAAAGTCAGCTCG-3′ and reverse 5′-GGTCTTGTTTCCTGCACTTC-3′ and GAPDH: forward 5′-GATGGGTGTGAAACCACGAGAAA-3′ and reverse 5′-ACGGATACATTGGGGGTAGGA-3′. The relative expression of IGF-1 was calculated by the 2^−ΔΔCt^ method (20).

### Hepatic metabolomic analysis

A total of 20 rats were used for hepatic metabolomic analysis, including 10 dw/+ rats (five females and five males) and 10 dw/dw rats (five females and five males). Liver tissues (100 mg) were individually ground with liquid nitrogen and the homogenate was resuspended with prechilled 80% methanol by vortexing well. The livers were incubated on ice for 5 min and then centrifuged at 15,000 ***g***, 4°C for 20 min. Some of the supernatant was diluted to a final concentration containing 53% methanol by LC-MS grade water. The samples were subsequently transferred to a fresh Eppendorf tube and then centrifuged at 15,000 ***g***, 4°C for 20 min. Finally, the supernatant was injected into the LC-MS/MS system for analysis. Samples were injected onto a Hypesil Gold column (100 × 2.1 mm, 1.9 μm) using a 17-min linear gradient at a flow rate of 0.2 mL/min. The eluents for the positive polarity mode were eluent A (0.1% FA in water) and eluent B (methanol). The eluents for the negative polarity mode were eluent A (5 mM ammonium acetate, pH 9.0) and eluent B (methanol). The solvent gradient was set as follows: 2% B, 1.5 min; 2–100% B, 3 min; 100% B, 10 min; 100–2% B, 10.1 min; 2% B, 12 min. The Q Exactive^TM^ HF mass spectrometer was operated in positive/negative polarity mode with a spray voltage of 3.5 kV, capillary temperature of 320°C, sheath gas flow rate of 35 psi, and auxiliary gas flow rate of 10 L/min. The S-lens RF level was set to 60, and the Aux gas heater temperature of 350°C.

The raw data files generated by UHPLC-MS/MS were processed using Compound Discoverer 3.1 (CD3.1, Thermo Fisher) to perform peak alignment, peak picking, and quantitation for each metabolite. These metabolites were annotated using the KEGG database, HMDB database, and LIPID Maps database. Principal component analysis (PCA) and partial least squares discriminant analysis (PLS-DA) were performed using metaX (a flexible and comprehensive software for processing metabolomics data). We applied univariate analysis (*t*-test) to calculate the statistical significance (*P*-value). The metabolites with VIP >1, *P* value <0.05, and fold change ≥1.5 or FC <0.667 were all considered to be differential metabolites. The volcano plot and heatmaps were generated using the ggplot2 and pheatmap package in R software, respectively. The correlation between differential metabolites was analyzed by corrplot (X) in R language (method = Pearson). The functions of these metabolites and metabolic pathways were studied using the KEGG database.

### Statistical analysis

Data were presented as mean ± s.e.m. The *t-*test was used for data analysis, and the Mann–Whitney *U* test was used when the data were not normally distributed. Correlations between metabolites and bone microstructural parameters were analyzed using Spearman’s correlation tests. All statistical computations were performed using SPSS version 22.0 (SPSS Inc). The significance level was set at *P* < 0.05.

## Results

### The reduced body weights and IGF-1 levels, and increased AST and ALT levels in dw/dw rats compared with dw/+ rats

**Figure 1:**
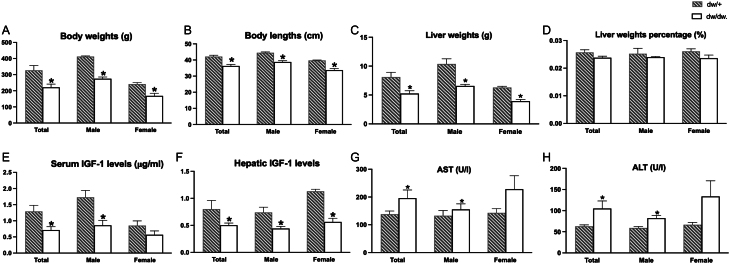
Clinical characteristics and serum biochemical parameters of dw/+ rats and dw/dw rats. (A) body weights, (B) body lengths, (C) liver weights, (D) liver weights percentages, (E) serum IGF-1 levels, (F) hepatic IGF-1 mRNA levels, (G) serum AST levels, and (H) serum ALT levels. *P < 0.05 vs dw/+ rats.

As shown in Fig. 1A and B, the body weights of total dw/dw rats, including males and females, showed a 33.8% decrease when compared to total dw/+ rats (221.66 ± 62.77 vs 334.92 ± 90.86 g, *P* < 0.05). The body lengths of total dw/dw rats were also decreased by 14.0% compared to total dw/+ rats (36.26 ± 3.22 vs 42.16 ± 2.61 cm, *P* < 0.05). Similar changes in body weights and body lengths also existed when analyzed by gender, as presented in Fig. 1A and B (*P* < 0.05). In addition, we also found that liver weights in total dw/dw decreased by 35.2% when compared with total dw/+ rats (Fig. 1C). The similar changes were also observed in males and females. However, there were no significant differences in liver weight percentages between dw/+ and dw/dw rats (Fig. 1D).

In our study, total dw/dw rats presented lower serum IGF-1 levels compared to total dw/+ rats, as shown in Fig. 1E (0.71 ± 0.31 vs 1.10 ± 0.64 μg/mL, *P* < 0.05). This phenomenon was observed in males but not females. Consistently, hepatic IGF-1 mRNA levels in total dw/dw rats were lower than those in dw/+ rats (Fig. 1F). This phenomenon still existed when analyzed by gender (*P* < 0.05). Besides, as shown in Fig. 1G and H, serum AST and ALT levels in total dw/dw rats exhibited 41.5% and 66.7% increases when compared to total dw/+ rats, respectively. Similar changes were also observed in male dw/dw rats.

### Hepatic steatosis and increased inflammatory cells in the livers of dw/dw rats compared with dw/+ rats by H&E staining

**Figure 2:**
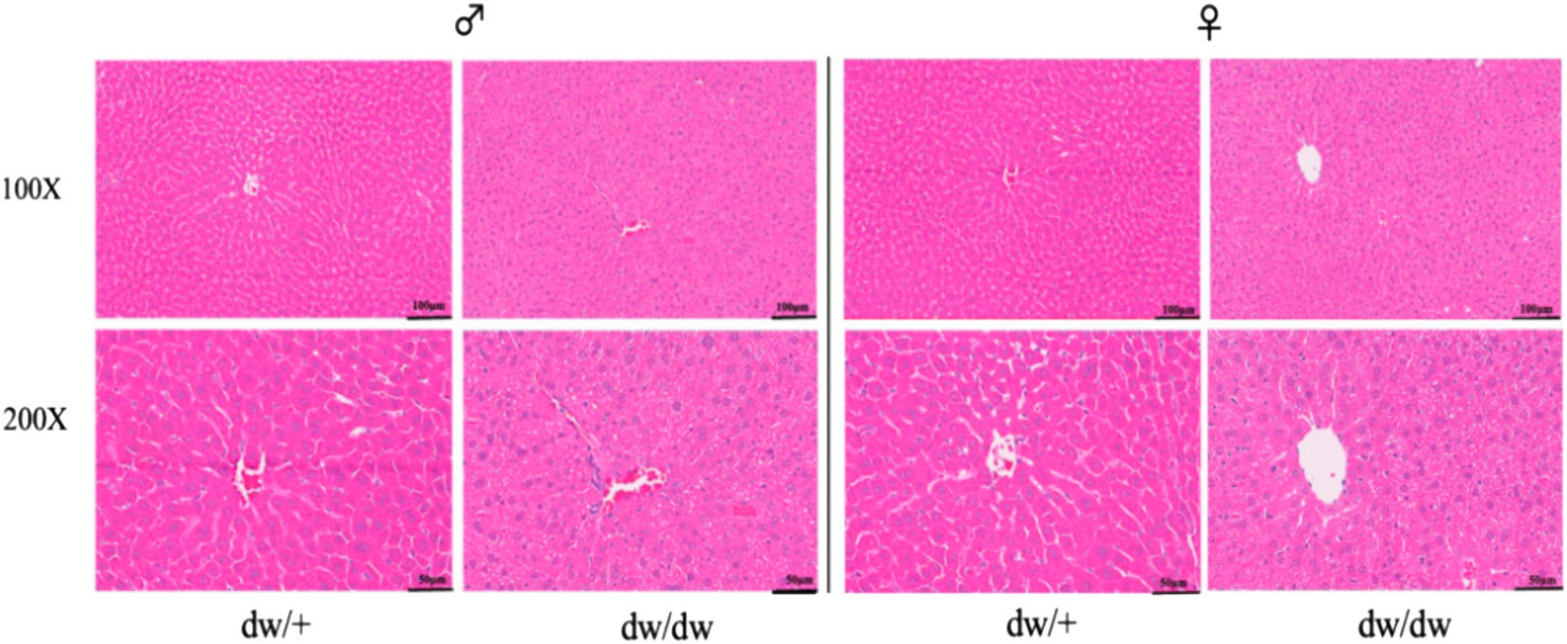
HE staining of liver tissue between dw/+ rats and dw/dw rats in males and females. Photographs were taken at 100× and 200× magnification.

As shown in Fig. 2, there were more lipid droplets in the HE staining of liver tissue in male dw/dw rats when compared to male dw/+ rats, indicating liver steatosis. Additionally, more inflammatory cell infiltration was observed in male dw/dw rats. The similar histological changes were also observed in females.

### Hepatic metabolomics

#### The screening for differential metabolites between dw/dw and dw/+ rats

**Figure 3:**
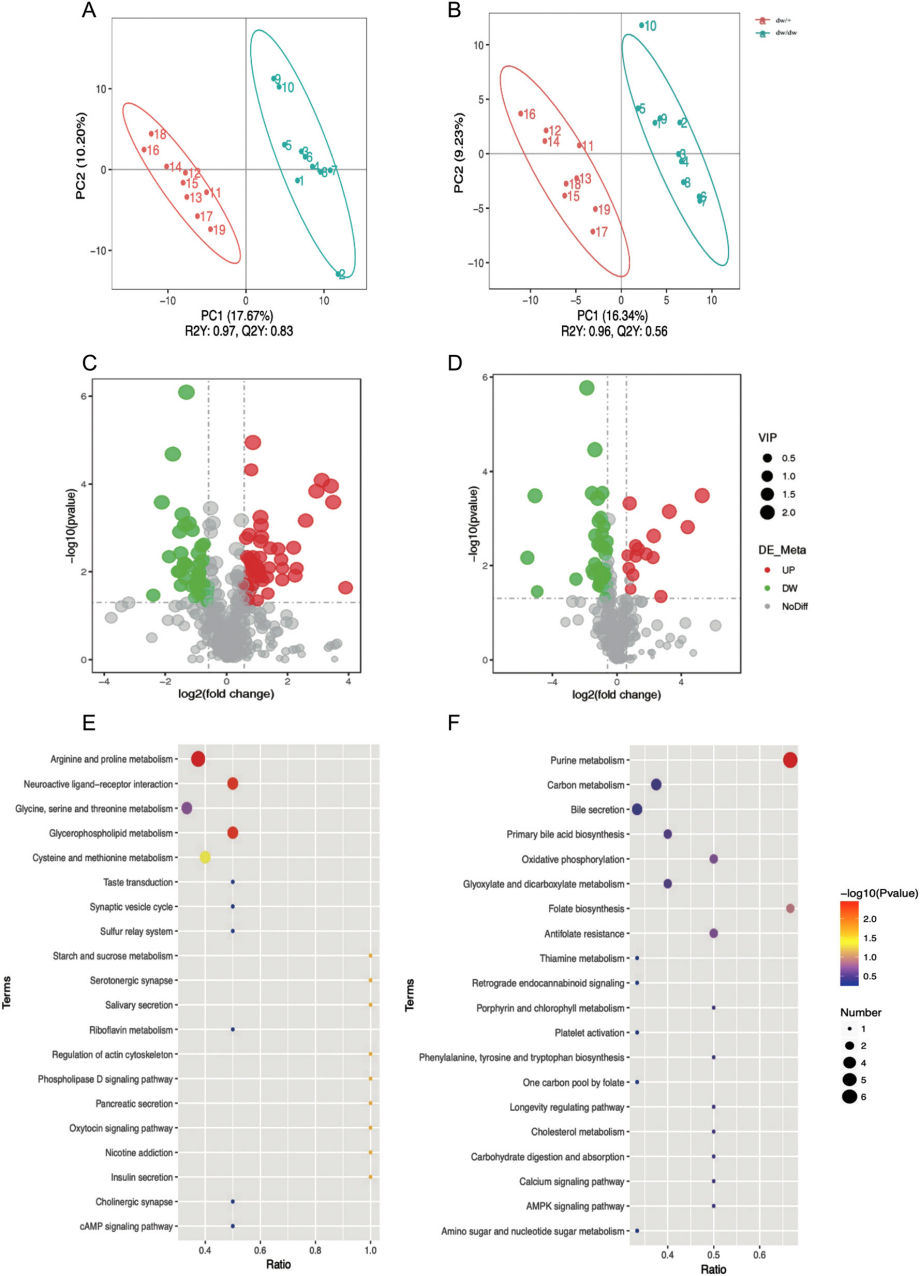
The untargeted hepatic metabolomics analysis between dw/+ and dw/dw rats. The screening for differential metabolites in the livers of dw/+ and dw/dw rats in positive (A) and negative (B) modes. Volcano maps of differential metabolites between dw/dw and dw/+ rats in positive (C) and negative modes (D). The KEGG pathway analysis of the differential metabolites in the livers of dw/+ and dw/dw rats in positive (E) and negative (F) ion modes.

As presented in Supplementary Fig. 1 (see section on supplementary materials given at the end of this article), the Pearson correlations (*R*^2^) between quality control (QC) samples were close in both positive and negative ion modes with a good reproducibility of QC, suggesting that the platform had essential stability throughout the analytical run. There was a good visual separation between dw/dw and dw/+ rats both in positive (Fig. 3A) and negative modes (Fig. 3B). A permutation test confirmed the validity of the model, with *R*^2^*Y* = 0.97, *Q*^2^*Y* = 0.83 in positive ion mode (Fig. 3A), and *R*^2^*Y* = 0.96, *Q*^2^*Y* = 0.75 in negative ion mode (Fig. 3B). Next, the PLS-DA-based approach was applied to maximize the differences and to screen the key metabolites. The significant metabolites were identified using VIP values (VIP > 1), *P* values (*P* < 0.05), and fold change (FC) ≥1.5 or FC <0.667. The characteristics of differential metabolites between dw/dw and dw/+ were detailed in Supplementary Table 1A (in positive ion mode) and 1B (in negative ion mode). As shown in Supplementary Table 1A and Fig. 3C, a total of 88 differential metabolites in positive ion mode were identified. Among them, 51 differential metabolites such as dehydrocholic acid, 7-ketodeoxycholic acid, and palmitoyl acid were elevated, while 37 differential metabolites, such as LPC 16:2, LPC18:3, LPC 22:6, S-adenosylmethionine, 4-guanidinobutyric acid, and homoarginine were decreased in dw/dw rats compared to dw/+ rats. As displayed in Supplementary Table 1B and Fig. 3D, a total of 51 differential metabolites in negative ion mode were identified. Among them, 15 differential metabolites such as adenosine diphosphate ribose, maltotriose, and 6-sialyllactose were increased, while 36 differential metabolites such as FAHFA 18:1, D-ribulose 1,5-bisphosphate, prostaglandin H2, and lactobionic acid were decreased in dw/dw rats.

#### KEGG pathway analysis of the differential metabolites between dw/dw and dw/+ rats

The metabolic pathway of related differential metabolites was analyzed between dw/dw rats and dw/+ rats in the KEGG database (Fig. 3E and F). According to the *P* value and the number of involved differential metabolites, the color of the points represented the *P* value of the hypergeometric test, and the size of the point represented the number of differential metabolites in the corresponding pathway. A larger point size indicated a higher concentration of metabolites in the pathway. As demonstrated in Fig. 3E (in positive ion mode) and F (in negative ion mode) between dw/dw and dw/+ rats, the top 20 pathways with the highest number of metabolites were statistically identified. Among them, the top five most enriched pathways in positive ion mode were the arginine and proline metabolism, neuroactive ligand–receptor interaction, glycine, serine, and threonine metabolism, glycerophospholipid metabolism, cysteine and methionine metabolism. The top five most enriched pathways in negative ion mode were purine metabolism, carbon metabolism, bile secretion, primary bile acid biosynthesis, and oxidative phosphorylation.

#### The relations between differential metabolites and phenotypes

**Figure 4:**
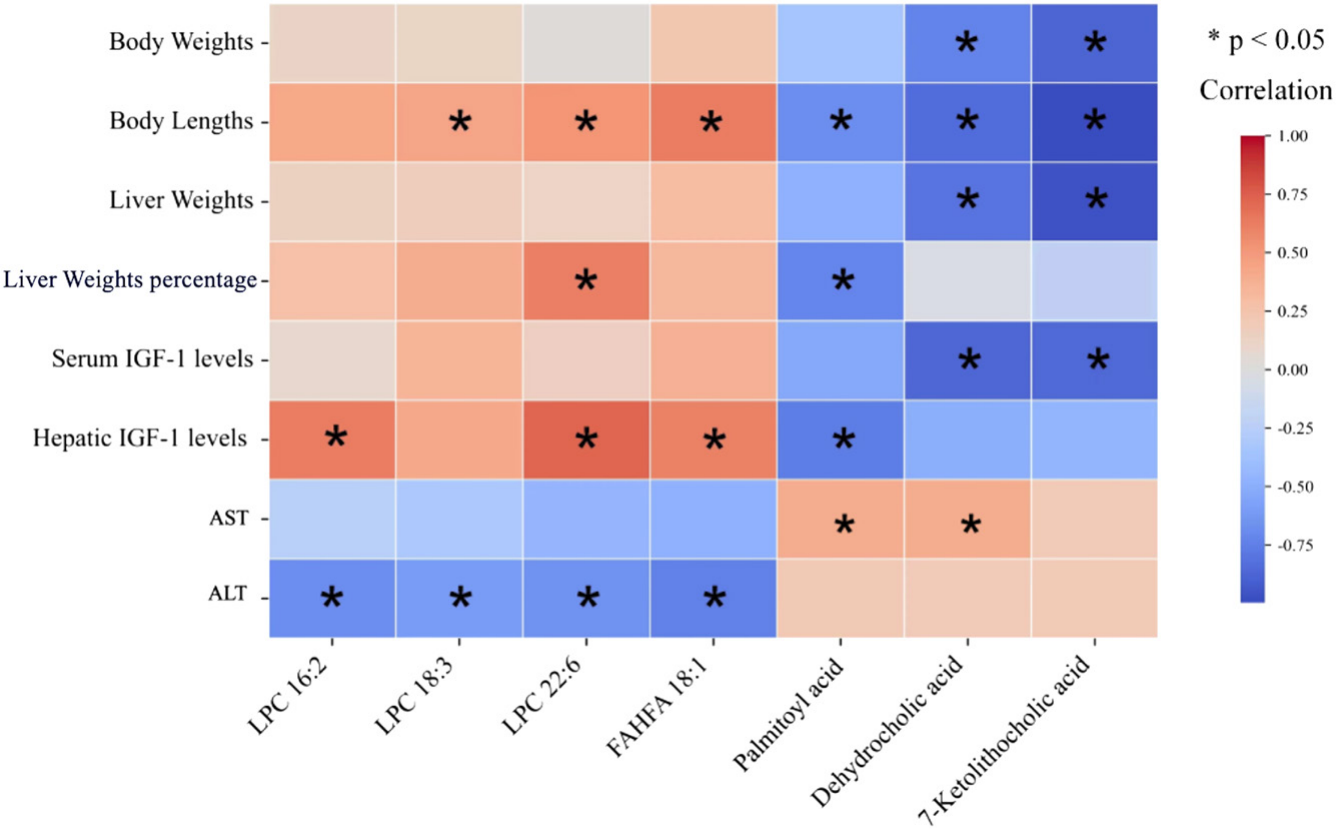
Correlation between differential metabolites and phenotypes in dw/+ rats and dw/dw rats.

To comprehensively analyze the relationship between the phenotypes and differential metabolites, Spearman’s correlation analysis was further performed. As shown in Fig. 4, LPC 16:2, LPC 18:3, LPC 22:6, and FAHFA 18:1 were significantly negatively associated with serum ALT levels, while palmitoyl acid and dehydrocholic acid were positively associated with serum AST levels (all *P* < 0.05). Moreover, dehydrocholic acid and 7-ketolithocholic acid were negatively related to body weights, liver weights, and serum IGF-1 levels (all *P* < 0.05). In addition, we also found that body lengths were positively related to LPC 18:3, LPC 22:6, and FAHFA 18:1, while they were negatively related to palmitoyl acid, dehydrocholic acid, and 7-ketolithocholic acid (all *P* < 0.05). Additionally, hepatic IGF-1 levels showed a positive correlation with LPC 16:2, LPC 22:6, and FAHFA 18:1, and a negative correlation with palmitoyl acid (all *P* < 0.05).

## Discussion

In our present study, we first performed untargeted hepatic metabolomics in congenital isolated GH-deficient Lewis dw/dw rats to explore the characterizations of hepatic metabolic profiles and their relationship with liver function. We found that (1) dw/dw rats represented reduced serum IGF-1 levels, increased AST and ALT levels, and more obvious hepatic steatosis compared with dw/+ rats; (2) a total of 88 and 51 metabolites were identified in our study in positive and negative ion modes, respectively. The most enriched pathways were the arginine and proline metabolism pathways, as well as bile secretion pathways; (3) there were significant associations between differential metabolites and abnormal phenotypes.

As is well known, the GH/IGF-1 axis is a crucial regulator of bone homeostasis throughout our life (21). It can determine longitudinal bone growth and skeletal maturation during adolescence and maintain bone mass in adults (21). Patients with congenital GHD exhibited significant retardation of growth and short stature in children due to the reduction of GH and IGF-1 levels (22). Consistently, we found that dw/dw rats represented lower body weights and lengths compared with dw/+ rats due to the lack of GH in somatic growth. Besides, we found that dw/dw rats showed impaired liver function accompanied by increased AST and ALT levels and hepatic steatosis. Consistent with our study, Diniz *et al*. found that congenital IGHD patients had higher ALT levels, an elevated prevalence of NAFLD, and increased hepatic steatosis (HS), but without advanced NAFLD forms when compared with the control group (4). Oliveira *et al.* reported that the size of the livers in IGHD patients was smaller than the control group with lower liver right lobe and left lobes (5). Besides, some studies showed that NAFLD is associated with low IGF-1 levels and IGF-1 levels are an independent prognostic factor for liver steatosis and NASH (2). It has been demonstrated that IGF-1 could improve the rat model of liver cirrhosis by reducing oxidative mitochondrial damage, correcting impaired mitochondrial function, and reducing caspase activity (23). Thus, we assumed that the decreased IGF-1 levels might contribute to the alterations in the indexes of hepatic functions and may relate to the impaired liver in congenital IGHD.

To the best of our knowledge, this is the first time that hepatic metabolomics methodology has been employed to investigate the hepatic metabolic profiles and their relationships with liver function. As a result, a total of 88 differential metabolites and 51 metabolites were identified in positive and negative ion modes, respectively. In our study, three metabolites, including LPC 16:2, LPC 18:3, and LPC 22:6, were significantly decreased in dw/dw rats and were negatively associated with ALT levels. Two metabolites, including LPC 18:3 and LPC 22:6, were positively related to body lengths. As a crucial marker of metabolic disruption, accumulating evidence has shown that LPC was associated with multiple liver diseases, such as hepatic cellular cancer (HCC), cirrhosis, and non-alcohol steatohepatitis (NASH) (24). For instance, it has been reported that the LPC (16:0/18:0/18:2) was significantly decreased in patients with cirrhosis and alcoholic liver cirrhosis, which was regarded as a biomarker for cirrhosis (24). Cantoni *et al.* reported that patients with chronic liver disease exhibited lower LPCs, which may be due to the inhibition of plasma enzyme lecithin acyltransferase (LCAT) synthesized by the liver (25). Our previous result also showed that LPC(15:0/0:0) levels were reduced in AGHD patients and LPCs were associated with metabolic disorders in AGHD patients. Besides, several studies found that LPC deficiency could cause cell death and promote lipid peroxidation and free radical damage, leading to the accumulation of large amounts of lipids in the liver, which might further explain the relationships between reduced LPC and abnormal liver function (26).

Fatty acid esters of hydroxy fatty acids (FAHFA) were a newly discovered class of endogenous mammalian lipids (27). It has been reported that FAHFA exhibits multiple beneficial metabolic effects, including anti-inflammatory effects and enhancing insulin-stimulated glucose transport, glucose-stimulated GLP-1, and insulin secretion (27, 28). Some studies have shown that patients with insulin resistance exhibited lower levels of palmitic acid-hydroxy stearic acid (PAHSA), a member of the FAHFA family (29). A study of animal experiments also revealed that FAHFA level was strongly decreased in high-fat diet (HFD) mice (28). Besides, it has been reported that PAHSA administration in mice can lower ambient glycemia, improve glucose tolerance, and reduce adipose tissue mass, suggesting that FAHFA can ameliorate metabolic disorders (28). In our study, FAHFA 18:1 level in dw/dw rats was lower and was negatively correlated to serum ALT levels and positively related to body lengths. Therefore, our study first found that decreased FAHFA levels were not only related to abnormal glucose and lipid metabolism but also associated with impaired liver function in GH deficiency conditions.

Besides, in contrast to the decreased alterations in four metabolites, including LPC 16:2, LPC 18:3, LPC 22:6, and FAHFA 18:1, we also found that three metabolites, including palmitoyl acid, dehydrocholic acid, and 7-ketodeoxycholic acid, were significantly increased in dw/dw rats. Palmitoyl acid, an amino acid produced from lysine and methionine, facilitates the uptake of fatty acids into the mitochondria for β-oxidation, which has been well-characterized as a mediator of hepatic lipotoxicity (30). Accumulating evidence has revealed that palmitoyl acid can activate proapoptotic signaling and trigger oxidative stress, leading to hepatocellular death and hepatic inflammation during the progression of NASH (31, 32, 33). A clinical trial has shown that a reduction in liver steatosis was associated with a decrease in serum palmitoyl acid (34). Severe hepatic inflammation was also observed in mice fed with palmitic acid supplements compared to controls (35). In our study, we first found that palmitoyl acid levels in the liver of dw/dw rats were higher than those in dw/+ rats and were positively related to serum AST levels. Thus, we assumed that the elevated palmitoyl acid level may be associated with abnormal hepatic function in dw/dw rats.

Bile acids, the general term for cholic acid in bile, are liver-derived metabolites of cholesterol and have a multitude of biological effects relevant to non-alcoholic fatty liver (NAFL) biology, including modulation of hepatic lipogenic drive, cell injury, inflammation, and fibrosis (36). Deoxycholic acid (DCA), a type of bile acid including dehydrocholic acid and 7-ketodeoxycholic acid, was reported to increase with liver disease activity and fibrosis stage (37). In our study, we found that dehydrocholic acid and 7-ketodeoxycholic acid were obviously elevated in dw/dw rats, and bile secretion was significantly enriched in KEGG pathway analysis, and dehydrocholic acid was positively related to serum AST levels. Consistent with our study, it has been reported that DCA was associated with advanced fibrosis. DCA increased in patients with fibrosis progression and declined in those with fibrosis regression (38, 39). Similar results were reported in animal experiments. It has been found that blocking DCA production can efficiently prevent HCC development in obese mice, and elevated DCA levels can inhibit hepatic genes with the consequences of the progression of NAFLD in sucralose-consuming mice (40, 41). Thus, we anticipate that bile acids might be associated with impaired hepatic function in dw/dw rats.

According to metabolite enrichment analysis, we found that significantly different metabolites between dw/dw and dw/+ rats, such as S-adenosylmethionine, 4-guanidinobutyric acid, and homoarginine, were well enriched in arginine and proline metabolism. It was well known that arginine and proline were mainly metabolized in the liver, and any liver injury might cause the disorder of arginine and proline metabolism (42, 43). Besides, arginine, the component of arginine vasopressin (AVP), plays a pivotal role in regulating blood pressure. It has been demonstrated that abnormal AVP levels could severely promote portal hypertension, which can lead to decreased liver function and hepatic encephalopathy (44, 45, 46). Thus, we speculate that arginine and proline metabolism might be associated with impaired hepatic function. In addition, it was reported that arginine can inhibit somatostatin and thus stimulate GH release from the pituitary gland, contributing to the fact that the peak GH levels could be assessed by arginine stimulation testing in clinical work (47). A systematic review of metabolomics in 362 patients with acromegaly from four electronic databases showed that arginine and proline were the most altered pathways (48). Therefore, we assumed that the abnormal GH levels might be associated with arginine metabolism, thus arginine metabolism was well enriched in dw/dw rats when compared with dw/+ rats.

## Conclusion

In conclusion, untargeted hepatic metabolomics in dw/dw rats revealed congenital isolated AGHD-specific metabolic patterns. The changes in metabolites and metabolic pathways may provide a molecular basis for hepatic steatosis in dw/dw rats. The study might provide new evidence to deepen our understanding of the underlying hepatic mechanisms in congenital isolated AGHD and give new clues in the hepatic treatment of patients with congenital isolated AGHD.

## Supplementary Materials

Figure S1: Pearson correlation between QC samples in positive (a) and negative (b) ion modes.

Table S1A: Identification of characteristic hepatic metabolites between dw/dw and dw/+ rats in positive ion mode

## Declaration of interest

The authors declare that there is no conflict of interest that could be perceived as prejudicing the impartiality of the study reported.

## Funding

This work was supported by the National Natural Science Foundation of Chinahttp://dx.doi.org/10.13039/501100001809 (Nos. 82270913 and 81970678), the Beijing Natural Science Foundation (No. 7222137), the CAMS Innovation Fundhttp://dx.doi.org/10.13039/100017413 for Medical Sciences (CIFMS) (2022-I2M-2-002), National High-Level Hospital Clinical Research Funding (No. 2022-PUMCH-B-016), National Key Clinical Specialty Capacity Improvement Project and the PUMCH Foundationhttp://dx.doi.org/10.13039/100001027 (pumch-2013-020).
