# Supplementary figures and images for: The distinct hepatic metabolic profile and relation with impaired liver function in congenital isolated growth hormone-deficient rats
Source: Endocr Connect. 2024 Apr 4;13(5):e230462. doi: 10.1530/EC-23-0462 (PMC11046350; doi:10.1530/EC-23-0462)

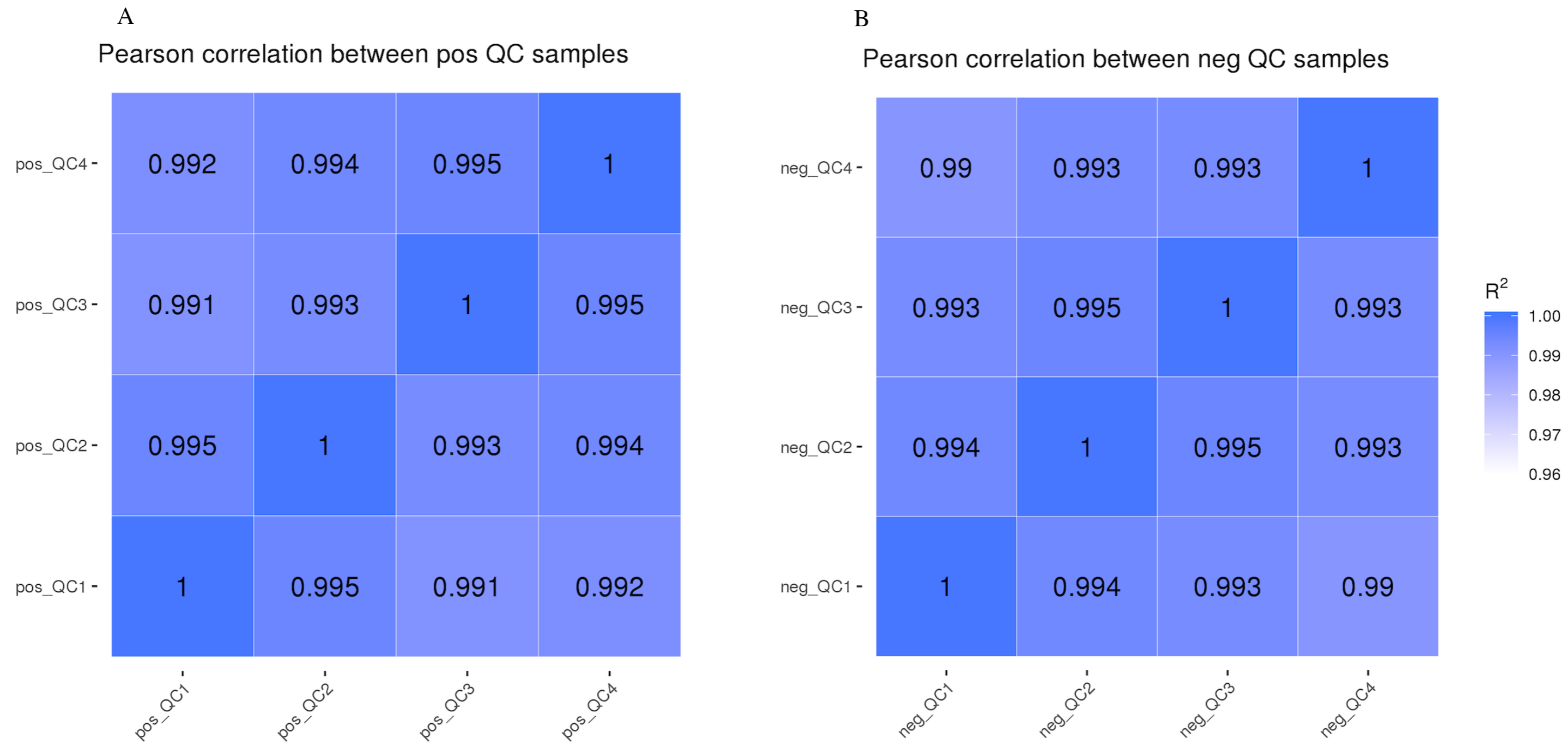

**Figure S1 : Pearson correlation between QC samples in positive (a) and negative (b) ion modes.**

Supplement: Figure S1: Pearson correlation between QC samples in positive (a) and negative (b) ion modes. [file supplementary_figure_1.pdf]
