# Supplementary material for: The distinct hepatic metabolic profile and relation with impaired liver function in congenital isolated growth hormone-deficient rats
Source: Endocr Connect. 2024 Apr 4;13(5):e230462. doi: 10.1530/EC-23-0462 (PMC11046350; doi:10.1530/EC-23-0462)
Supplement: Table S1A: Identification of characteristic hepatic metabolites between dw/dw and dw/+ rats in positive ion mode [file supplementary_table_1.pdf]

Table S1A:
Identification of characteristic hepatic metabolites between dw/dw and dw/+ rats in positive ion mode

**Compound ID**	**Name**	**FC**	**P value**	**VIP**
Com_1718_pos	Guanidinoethyl sulfonate	0.403431	0.000001	2.235373
Com_6221_pos	(±)8-HEPE	1.821774	0.000011	2.215393
Com_4545_pos	5'-S-Methyl-5'-thioadenosine	0.296574	0.000021	2.130018
Com_3110_pos	α-Lactose	7.697133	0.000146	2.110869
Com_1472_pos	Melezitose	10.678236	0.000111	2.097164
Com_2322_pos	Maltotetraose	8.696758	0.000083	2.061624
Com_451_pos	Trehalose	11.256538	0.000260	2.038273
Com_3187_pos	Prostaglandin F2alpha	2.165879	0.000568	2.003952
Com_2484_pos	Palmitoyl acid	1.425091	0.000782	1.981308
Com_7263_pos	ACar 18:0	0.230963	0.000262	1.974512
Com_1371_pos	2-hydroxy-6-[(8Z,11Z)-pentadeca-8,11,14-trien-1-yl]benzoic acid	2.187394	0.000874	1.949798
Com_1986_pos	1,5,8-Trihydroxy-9-oxo-9H-xanthen-3-yl beta-D-glucopyranoside	6.036744	0.000683	1.907745
Com_770_pos	S-Adenosylmethionine	0.367070	0.000489	1.894053
Com_2453_pos	Methenolone	2.205638	0.001598	1.881606
Com_28348_pos	ACar 17:1	0.530718	0.003781	1.846859
Com_3740_pos	5'-Deoxy-5'-(Methylthio)Adenosine	0.389499	0.000856	1.838838
Com_6170_pos	4-(2,3-dihydro-1,4-benzodioxin-6-yl)butanoic acid	1.672836	0.001434	1.777826
Com_299_pos	4-Guanidinobutyric acid	0.354695	0.003763	1.773800
Com_434_pos	Glucose 1-phosphate	2.174575	0.002029	1.768968

Com_2170_pos	12-Oxo phytodienoic acid	1.818165	0.004611	1.760996
Com_436_pos	D-(+)-Maltose	4.595602	0.002819	1.746025
Com_9179_pos	Nicotinamide adenine dinucleotide phosphate (NADP+)	0.346458	0.001227	1.700825
Com_2697_pos	Carnosine	4.744752	0.012230	1.692362
Com_1010_pos	ACar 18:1	0.384977	0.006422	1.689771
Com_553_pos	4-Guanidinobutanoic acid	0.414936	0.007557	1.644407
Com_11528_pos	FNK	0.570309	0.002603	1.639635
Com_2187_pos	Thymine	3.230204	0.003014	1.621536
Com_23_pos	Creatine	2.258425	0.015668	1.612065
Com_7217_pos	11-Deoxy prostaglandin F1α	0.462173	0.001132	1.601388
Com_537_pos	Taurocholic acid sodium salt hydrate	2.042670	0.010421	1.595935
Com_3893_pos	(-)-Epigallocatechin	0.579598	0.005520	1.595553
Com_722_pos	2-[(7-methyl-2,3-dihydro-1H-inden-4-yl)oxy]pyridin-3-amine	14.919803	0.023031	1.595428
Com_612_pos	ACar 18:2	0.540509	0.010172	1.565325
Com_4340_pos	Uracil 1-beta-D-arabinofuranoside	1.568596	0.001720	1.563338
Com_27868_pos	Bilirubin	0.642375	0.025406	1.555111
Com_46_pos	Tauro-alpha-Muricholic acid sodium salt	1.916331	0.013113	1.553119
Com_12059_pos	Gly-Tyr-Ala	2.709915	0.002903	1.553007
Com_3131_pos	(5E)-7-methylidene-10-oxo-4-(propan-2-yl)undec-5-enoic acid	0.392330	0.014175	1.531869
Com_18_pos	Acetylcholine	0.349346	0.009767	1.524496
Com_3729_pos	Ala-Val	2.135037	0.004681	1.514120
Com_73_pos	Benzoylecgonine	0.27248	0.0045	1.5004

		2	33	33
Com_7449_pos	Pyridoxal 5'-phosphate	0.598079	0.002350	1.491047
Com_7152_pos	PC (4:0/16:2)	2.510183	0.013209	1.486732
Com_11281_pos	3-hydroxy-2-[5-nitro-2-(1-pyrrolidinyl)benzyl]propanenitrile	1.601304	0.004534	1.484181
Com_3777_pos	N-lactoyl-phenylalanine	1.668238	0.004959	1.476622
Com_1253_pos	tert-Butyl N-[1-(aminocarbonyl)-3-methylbutyl]carbamate	3.456190	0.005265	1.472847
Com_15831_pos	AQH	1.749531	0.006418	1.456168
Com_692_pos	7-Ketodeoxycholic acid	1.190138	0.034018	1.450584
Com_4482_pos	PC (18:5e/2:0)	3.548000	0.015263	1.449641
Com_16_pos	Phosphocholine	0.333349	0.009843	1.447560
Com_33667_pos	PC (16:2e/2:0)	1.523111	0.018921	1.443803
Com_2536_pos	Dehydrocholic acid	1.355182	0.020273	1.438183
Com_2149_pos	LPC 18:3	0.811984	0.008415	1.434608
Com_7783_pos	LPC 16:2	0.756174	0.000048	1.418085
Com_3142_pos	Guanidinosuccinic acid	1.978362	0.007090	1.399654
Com_2818_pos	APK	0.544268	0.006555	1.399179
Com_4729_pos	ACar 22:6	0.538453	0.012848	1.390688
Com_4119_pos	2-[(3S)-1-Benzyl-3-pyrrolidinyl]-1,3-benzothiazole	2.089680	0.008556	1.382464
Com_412_pos	ACar 20:4	0.592803	0.019273	1.379605
Com_3841_pos	ACar 16:1	0.504687	0.020996	1.372478
Com_2614_pos	Homoarginine	0.588066	0.031079	1.368246
Com_45754_pos	L-Leucyl-L-alanine Hydrate	1.792285	0.006172	1.367845

Com_346_pos	β-Nicotinamide mononucleotide	0.506793	0.017547	1.367765
Com_2878_pos	2-(2-amino-3-methylbutanamido)-3-phenylpropanoic acid	4.919920	0.008320	1.363622
Com_7785_pos	Coenzyme Q2	0.538363	0.031583	1.359499
Com_3266_pos	PC (14:1e/3:0)	2.633882	0.008064	1.358186
Com_5211_pos	3-(1-benzylpiperidin-4-yl)-3H-[1,2,3]triazolo[4,5-b]pyridine	0.397183	0.006584	1.351191
Com_1042_pos	N-Acetylglucosamine 1-phosphate	0.454927	0.021237	1.350705
Com_5719_pos	(±)-Abscisic acid	0.523987	0.011248	1.306033
Com_2288_pos	Ala-Ile	2.177807	0.013246	1.289706
Com_81_pos	LPC 22:6	0.786444	0.009998	1.286196
Com_6872_pos	L-Cystathionine	1.625681	0.043203	1.272731
Com_189_pos	L-Cysteine-glutathione gisulfide	1.707741	0.026478	1.269933
Com_7496_pos	Asp-Phe methyl ester	1.784004	0.007804	1.251944
Com_250_pos	Stearamide	0.642037	0.031804	1.247835
Com_148_pos	Riboflavin	0.659295	0.021204	1.245821
Com_11121_pos	Choline Glycerophosphate	1.663555	0.011623	1.236199
Com_28217_pos	ACar 15:0	0.646752	0.046778	1.233916
Com_1925_pos	2-Arachidonoyl glycerol	3.566202	0.008290	1.232606
Com_2347_pos	(±)13-HpODE	1.525885	0.047118	1.232246
Com_11829_pos	Traumatic acid	1.541641	0.030656	1.230977
Com_4663_pos	ACar 18:3	0.514426	0.019777	1.228800
Com_2605_pos	Gly-Tyr	1.581866	0.020731	1.227529
Com_966_po	PC (14:1e/2:0)	2.56066	0.0311	1.2002

s		6	45	21
Com_3492_pos	Ala-Gln	1.632253	0.023212	1.172352
Com_3045_pos	INK	1.801577	0.020375	1.160818
Com_7519_pos	4-(3,4-dimethoxyphenyl)-3-methyl-1H-pyrazol-5-amine	0.486571	0.038221	1.141631
Com_5380_pos	Ala-Leu	2.021864	0.044569	1.109517

Table S1B:

Identification of characteristic hepatic metabolites between dw/dw and dw/+ rats in negative ion mode

Compound_ID	Name	FC	Pvalue	VIP
Com_1444_neg	D-Ribulose 1,5-bisphosphate	0.274887	0.000002	2.389839
Com_1996_neg	Lactobionic acid	0.389175	0.000035	2.323232
Com_721_neg	5-Phosphoribosyl 1-pyrophosphate	0.535582	0.000287	1.919539
Com_3512_neg	11-Deoxy prostaglandin F2	0.346017	0.000291	2.090066
Com_139_neg	Adenosine diphosphate ribose	39.244258	0.000325	2.263996
Com_458_neg	7-Ketolithocholic acid	0.029566	0.000328	2.220336
Com_4190_neg	Prostaglandin H2	0.437278	0.000370	2.342266
Com_52_neg	3'-Adenosine monophosphate (3'-AMP)	1.739557	0.000476	1.937305
Com_1653_neg	Maltotriose	9.535630	0.000710	2.163770
Com_1344_neg	Pyridoxal-5'-phosphate	0.503691	0.000961	1.796556
Com_2395_neg	NAD+	0.461640	0.001126	2.028628
Com_2612_neg	Deoxyguanosine	0.572794	0.001454	1.674929
Com_121_n	D-Raffinose	21.0493	0.0015	1.9575

eg		16	23	03
Com_1807_neg	Pyridoxal 5'-phosphate hydrate	0.528897	0.002058	1.606793
Com_3537_neg	Estradiol	0.396859	0.002300	1.973627
Com_2988_neg	6-Sialyllactose	4.885811	0.002335	2.027945
Com_208_neg	2-deoxyglucose-6-phosphate	0.657819	0.003080	1.653652
Com_3077_neg	2,3-dinor Prostaglandin E1	0.449526	0.003484	1.890968
Com_3461_neg	Tyrosylalanine	2.242995	0.003788	1.582399
Com_2205_neg	Hypotaurine	0.486439	0.003875	1.507783
Com_916_neg	Guanosine	0.583168	0.003976	1.282348
Com_882_neg	Reduced nicotinamide adenine dinucleotide	2.490638	0.004483	1.858252
Com_5242_neg	(1E)-1,7-bis(4-hydroxyphenyl)hept-1-en-3-one	0.660892	0.004713	1.164166
Com_2800_neg	N1-[1-(3-isopropenylphenyl)-1-methylethyl]-3-oxobutanamide	3.533302	0.005662	1.541631
Com_136_neg	N-Acetyl-D-glucosamine 1-phosphate	0.610606	0.005678	1.681843
Com_5639_neg	Ala-trp	1.572578	0.006047	1.157817
Com_3805_neg	Phe-Pro	2.249076	0.006317	1.481589
Com_2466_neg	Feruloyl Putrescine	4.740283	0.006744	1.552259
Com_39_neg	Deoxycholic Acid	0.021338	0.006820	1.903984
Com_396_neg	Citric acid	0.387177	0.009112	1.584618
Com_2599_neg	Capryloylglycine	0.470562	0.011181	1.707829
Com_2666_neg	Benzyl cinnamate	1.671392	0.011367	1.391671
Com_1764_neg	Taurochenodeoxycholic acid	0.582871	0.011738	1.470372
Com_641_neg	Dihydroroseoside	0.343424	0.012605	1.499885

Com_4997_neg	FAHFA 18:1	0.517437	0.012721	1.297538
Com_466_neg	Orotic acid	0.357875	0.014898	1.347535
Com_3491_neg	trans-10-Heptadecenoic Acid	0.543667	0.015409	1.519919
Com_5534_neg	Guanine	1.996688	0.015663	1.510769
Com_3822_neg	13,14-Dihydro prostaglandin E1	0.597853	0.016267	1.695213
Com_1681_neg	Stercobilin	0.568128	0.016380	1.520525
Com_944_neg	Chenodeoxycholic Acid	0.171666	0.019346	1.621592
Com_974_neg	3-Indoxyl sulphate	0.549680	0.019518	1.391418
Com_3494_neg	13,14-dihydro-15-keto-tetranor Prostaglandin F1	0.484264	0.020428	1.671298
Com_1199_neg	Adenine	0.647042	0.025057	1.596974
Com_2357_neg	9(Z),11(E)-Conjugated linoleic acid	0.552788	0.025669	1.572129
Com_5187_neg	7,8-Dihydrofolate	0.452276	0.026421	1.381380
Com_1106_neg	Dodecanedioic acid	0.496484	0.027720	1.471475
Com_1533_neg	Asp-Phe	1.815867	0.031373	1.078622
Com_230_neg	Glycolithocholic acid	0.032496	0.035395	1.343122
Com_1374_neg	L-Anserine	6.650191	0.045201	1.587412
Com_486_neg	4-(octyloxy)benzoic acid	0.602957	0.047020	1.098689
